# An In Vitro Comparison of the Digestibility and Gastrointestinal Fate of Scallops and Plant-Based Scallop Analogs

**DOI:** 10.3390/foods12152928

**Published:** 2023-08-02

**Authors:** Zhiyun Zhang, Dingkui Qin, Kanon Kobata, Jiajia Rao, Jiakai Lu, David Julian McClements

**Affiliations:** 1 Department of Food Science, University of Massachusetts, Amherst, MA 01003, USA; kevinchang0711@gmail.com (Z.Z.); dingkui@umass.edu (D.Q.); kkobata@umass.edu (K.K.); jiakailu@umass.edu (J.L.); 2 Department of Plant Science, North Dakota State University, Fargo, ND 58102, USA; jiajia.rao@ndsu.edu; 3 Department of Food Science & Bioengineering, Zhejiang Gongshang University, 18 Xuezheng Street, Hangzhou 310018, China

**Keywords:** plant-based food analogs, sustainability, seafood, in vitro digestion, bioavailability

## Abstract

Concerns exist regarding the negative environmental impact and health risks associated with ocean fishing and aquaculture, such as stock depletion, pollution, biodiversity loss, and toxin presence. To address these concerns, plant-based seafood analogs are being developed. Our previous study successfully created plant-based scallop analogs using pea proteins and citrus pectin, resembling real scallops in appearance and texture. This study focuses on comparing the digestive fate of these analogs to real scallops, as it can impact their nutritional properties. Using an in vitro digestion model (INFOGEST), we simulated oral, gastric, and small intestinal conditions. The analysis revealed differences in the microstructure, physicochemical properties, and protein digestibility between the plant-based scallops and real scallops. The particle size and charge followed the following similar trends for both types of scallops: the particle size decreased from the mouth to the stomach to the small intestine; the particles were negative in the mouth, positive in the stomach, and negative in the small intestine. The protein digestibility of the plant-based scallops was considerably lower than that of real scallops. For instance, around 18.8% and 61.4% of protein was digested in the stomach and small intestine phases for the real scallop (80.2% total digestion), whereas around 8.7% and 47.7% of the protein was digested for the plant-based scallop (56.4% total digestion). The lower digestibility of the plant-based scallops may have been due to differences in the protein structure, the presence of dietary fibers (pectin), or antinutritional factors in the plant proteins. These findings are crucial for developing more sustainable next-generation plant-based seafood analogs.

## 1. Introduction

Increasingly, consumers are recognizing the pressing need to transition from animal-based diets to plant-based ones, driven by concerns about the detrimental effects of livestock production and fishing on animal welfare, the environment, and human health [1,2]. The raising of livestock for human food is a major emitter of greenhouse gasses, pollutes the land, water, and air, requires large amounts of fresh water, promotes biodiversity loss, increases the risk of antibiotic resistance and zoonotic diseases, and requires the killing of billions of animals each year [3]. The seafood industry has also been reported to have major negative impacts on the global ecosystem. A Food and Agriculture Organization (FAO) report stated that about 90% of the species caught through wild fisheries are either overfished or fished at their maximal capacity [4]. Moreover, global CO_2_ emission from fuel consumption by the seafood industry has increased over four-fold over the past seven decades, reaching 159 million tons of CO_2_ in 2016 [5]. There are also some health concerns about consuming certain kinds of seafood due to the presence of heavy metals and other toxic contaminants [6,7]. Seafood consumption in the United States is responsible for approximately 90% of the mercury found in the human body. Governments are also introducing new legislation to limit the types and quantities of certain seafood species that can be captured and consumed. Consequently, there is growing interest in shifting towards more sustainable and healthy alternatives to conventional seafood, including plant-based seafood analogs [8]. Increased consumption of these plant-based products may help to address environmental concerns (stock depletion, pollution, and biodiversity loss) and health concerns (bioaccumulated toxins, norovirus, and allergies) associated with conventional seafood [9]. Nevertheless, it is important that these foods be designed so that they are nutritionally similar or better than conventional seafood, so adopting a more plant-based diet does not result in any negative long-term health effects [10,11].

Extrusion and shearing technologies are commonly used to produce plant-based meat and seafood analogs [12,13,14]. Nevertheless, these processing operations require specialized equipment and involve substantial energy inputs, thereby limiting their suitability for small-sized companies and raising environmental concerns [15]. Soft matter physics approaches can also be utilized to assemble meat and seafood analogs from plant-derived ingredients [16,17,18,19]. These approaches are based on the fact that mixtures of biopolymers (usually proteins and polysaccharides) can spontaneously phase separate if there are strong enough attractive or repulsive interactions amongst them. Phase separation is influenced by various factors that require careful control, including the type and concentration of biopolymers, pH, ionic strength, and temperature. Once phase separation occurs, it results in the formation of a “water-in-water” (W/W) emulsion, where one biopolymer dominates the dispersed phase while the other biopolymer dominates the continuous phase. These W/W emulsions possess a low interfacial tension, enabling them to easily deform and elongate into fiber-like structures under low shear stresses. These fiber-like structures can then be solidified by inducing gelation in either the dispersed or continuous biopolymer phases, or both.

In our previous study, we created plant-based scallop analogs using a soft matter approach [18]. Briefly, heat-denatured pea protein and citrus pectin were mixed together under conditions where phase separation occurred. The resulting system was then sheared to form fibers, placed in a mold, and crosslinked by adding transglutaminase to crosslink the proteins and set the fiber structures [18]. Pea protein and citrus pectin were selected as structuring ingredients to form the scallop analogs because of their abundance, affordability, and functionality. The protein content of these plant-based scallops was formulated to closely resemble that of real scallops, aiming to achieve a closer match in terms of their nutritional properties. Nevertheless, plant proteins generally exhibit lower digestibility than animal proteins, which is attributed to differences in their molecular structures and the presence of antinutritional factors commonly found in edible plants [10,11,20]. Hence, it is crucial to assess the digestibility of plant-based scallops in comparison to real scallops, as it can significantly impact their implications for human nutrition and health.

Based on the variations in their compositions and structures, we postulated that there would be divergences in the gastrointestinal fate of real scallops and plant-based scallop analogs. To investigate this, we employed a standardized in vitro digestion model (INFOGEST) that simulates the conditions of the mouth, stomach, and small intestine in the human gastrointestinal tract (GIT) to compare the gastrointestinal fates of these two scallop types [21,22]. The plant-based scallops were fortified with ω-3 fatty acids (flaxseed oil) so as to match the lipid profile of real scallops. The structural, physicochemical, and digestibility properties of both scallops and scallop analogs were tracked during their progression through simulated gastrointestinal conditions. This information can aid in the development of plant-based seafood products with enhanced nutritional attributes.

## 2. Materials and Methods

### 2.1. Materials

Native yellow pea flour was generously provided by Prof. Jiajia Rao’s lab at North Dakota State University. It has been reported that the ash, crude protein, lipid, and starch contents of this pea flour is in the range 2.2–3.1%, 22.5–28.7%, 1.0–3.4%, and 43.6–49.6%, respectively [23]. The proteins were extracted from this flour using a previously described method [18]. Citrus peel pectin (galacturonic acid ≥ 74.0% dried basis) was obtained from Sigma-Aldrich Co., Ltd. (St. Louis, MO, USA). The food-grade crosslinking enzyme transglutaminase (ACTIVA RM, T-gase, Hamburg, Germany) was purchased from Ajinomoto North America., Inc. (Chicago, IL, USA). Raw sea scallops were acquired from a local grocery store (Stop & Shop, Amherst, MA, USA) and stored in a freezer at −20 °C until use (within one week). Sodium hydroxide (NaOH) and hydrochloric acid (HCl) were obtained from Fisher Scientific (Waltham, MA, USA). Nile red and Fluorescein isothiocyanate (FITC), lipid and protein fluorescence stains, were purchased from Sigma-Aldrich (St. Louis, MO, USA). The protein measurement was conducted using the Bradford reagent assay obtained from Bio-Rad (Hercules, CA, USA). All aqueous solutions were prepared using double distilled water obtained from a purification unit.

### 2.2. Flaxseed Emulsion Production

To incorporate a plant-based source of ω-3 fatty acids into the scallop analogs, flaxseed oil-in-water emulsions were fabricated. Initially, 1% (*w*/*w*) pea protein powder was dissolved in sodium phosphate buffer overnight at 4 °C. The pH of this mixture was then adjusted to 7.0 using NaOH or HCl solutions. Coarse oil-in-water emulsions were created by blending the oil phase (10% *w*/*w*) and aqueous phase (90% *w*/*w*) using a high-shear mixer (M133/1281-0, Biospec Products Inc., Bartlesville, OK) for 2 min at 9000 rpm. Subsequently, this coarse emulsion underwent three passes through a microfluidizer (Microfluidics, Newton, MA, USA) operating at 12,000 psi to produce a fine emulsion. (*It should be noted that coarse flaxseed emulsions could be incorporated into scallop analogs if a microfluidizer was not available*).

### 2.3. Scallop Analog (Pea Protein–Pectin Gel) Production

Plant-based scallops were created using a method described in our previous study [18]. To prepare pea protein stock solutions (20% *w*/*w*), extracted pea proteins were rehydrated overnight. These stock solutions were subsequently diluted to a protein concentration of 10% *w*/*w* and adjusted to pH 7.0. In a 30 mL beaker serving as a scallop-shaped mold, 9.5 g of the pea protein solution and 0.5 g of flaxseed oil emulsion (obtained from Section 2.2) were combined and mixed. The resulting solution was then heated to 95 °C for 30 min to induce protein denaturation and aggregation. After cooling the heat-denatured pea proteins and pea protein-coated oil droplets in an ice bath for 30 min, pectin (0.5% w_pectin_/w_total_) was added, and the mixtures were stirred at 500 rpm for 60 min at room temperature to ensure dissolution. Transglutaminase (T-gase, 2% w_T-gase_/w_total_) was subsequently added, and the mixture was stirred for 30 min at 500 rpm using a magnetic stirrer at room temperature to facilitate enzyme dissolution. The stir bar was then removed, and the samples were incubated at 50 °C for 30 min to promote protein crosslinking, followed by 30 min in an ice bath. The resulting gels were gently removed from the beakers and placed onto Petri dishes.

### 2.4. In Vitro Digestion

The in vitro digestion of the scallops was characterized using the INFOGEST method, which has been described in detail previously [21,24]. Briefly, this method involved passing the scallops through simulated oral (pH 7.0, mucin, amylase, 2 min), gastric (pH 3.0, pepsin, 120 min), and small intestinal (pH 7.0, bile salts, trypsin, lipase, 120 min) phases. The details of the compositions of the gastrointestinal fluids used are provided in the INFOGEST references just mentioned. The only difference was that NaHCO_3_ was replaced with NaCl to avoid potential pH changes during storage and digestion in an open beaker (because sodium bicarbonate may react with oxygen to form carbonic acid). Before digestion, the scallop samples were fully cooked for 15 min as described previously [18]. All stock solutions were pre-heated to 37 °C prior to use and all digestion procedures were conducted at this temperature.

Protein hydrolysis in the gastric phase was followed using an automatic titration instrument (Metrohm, USA Inc., Riverview, FL, USA) by measuring the volume of HCl solution (50 mM) required to maintain the sample at pH 3.0 for 120 min.

### 2.5. Characterization of Physicochemical Properties

#### 2.5.1. Particle Dimensions and Surface Charge

The mean particle diameters and size distributions of the samples were analyzed after the mouth, stomach, and small intestine phases using laser diffraction (Mastersizer 2000, Malvern Instruments, Worcestershire, UK). From the particle size distributions, the surface-weighted (d_32_) and volume-weighted (d_43_) particle diameters were calculated. The surface potential (ζ-potential) of the digested sample particles was measured using an electrophoresis device (Nano-ZS, Malvern Instruments). Prior to the measurements, the samples were diluted with electrolyte solutions corresponding to the respective gastrointestinal phase (mouth, stomach, or small intestine) to an appropriate concentration to mitigate multiple scattering effects.

#### 2.5.2. Microstructure

The microstructures of the scallops and scallop analogs were characterized by fluorescence confocal scanning microscopy (Nikon D-Eclipse C1 80i, Nikon, NY, USA). Protein (FITC) and lipid (Nile Red) fluorescence stains were used to identify the lipids and proteins within the samples as described previously [24]. In particular, a protein-soluble stain (FITC, 30 μL) and fat-soluble stain (Nile Red, 20 μL) were mixed with 300 μL of sample (real or plant-based scallop). After staining, the samples were placed onto a glass microscope slide and then observed at 400× magnification.

### 2.6. Protein Hydrolysis

The degree of hydrolysis (DH) of the proteins in the gastric and small intestinal phases was determined using the pH-stat method, following a previously described procedure [24]:

(1) $$\mathrm{DH}=\frac{V_{t}\times C_{t}}{M_{protein}\times P}\times F_{PH}\times 100\%$$

Here, V_t_ and C_t_ are the volume and concentration of titrating solutions, respectively. In the gastric phase, V_t_ and C_t_ are the volume and molarity (0.1 M) of the HCl solution used to keep the system at pH 3.0. In the small intestinal phase, V_t_ and C_t_ are the volume and molarity (0.25 M) of the NaOH solution used to keep the system at pH 7.0. Here, M_protein_ is the mass of protein in either the gastric phase or the small intestinal phase. *P* is the moles of peptide bonds per gram of protein (mol/g protein), which was taken to be 7.6 for scallops and 7.8 for scallop analogs. F_pH_ is a constant that equals 1.8 under gastric conditions (pH = 3.0 and 37 °C) and 3.03 under small intestinal conditions (pH = 7.0 and 37 °C) [24].

### 2.7. Statistical Analysis

Each experiment conducted in this study was replicated at least three times. The results were then analyzed to calculate the means and standard deviations. Statistical differences (*p* < 0.01) between the samples were determined using ANOVA analysis, followed by post-hoc Tukey HSD Test.

## 3. Results and Discussions

### 3.1. Gastrointestinal Fate of Scallops and Scallop Analogs

The objective of these experiments was to investigate the potential differences in the gastrointestinal behavior of real scallops and plant-based scallop analogs. To achieve this, variations in their microstructures, physicochemical attributes, and digestion were assessed as they passed through the gastrointestinal model.

### 3.2. Particle Size Characteristics

The dimensions and morphology of the particles within a particular gastrointestinal region reflect the extent of the breakdown of solid foods due to mechanical forces, alterations in solution conditions (like pH, mineral composition, and bile acid concentration), and enzymatic digestion (like hydrolysis by proteases, lipases, and amylases). Thus, the particle size characteristics of each sample were measured. The properties of plant-based scallop analogs with and without ω-3 fatty acids (flaxseed oil droplets), as well as real scallops, were analyzed and compared.

**Figure 1:**
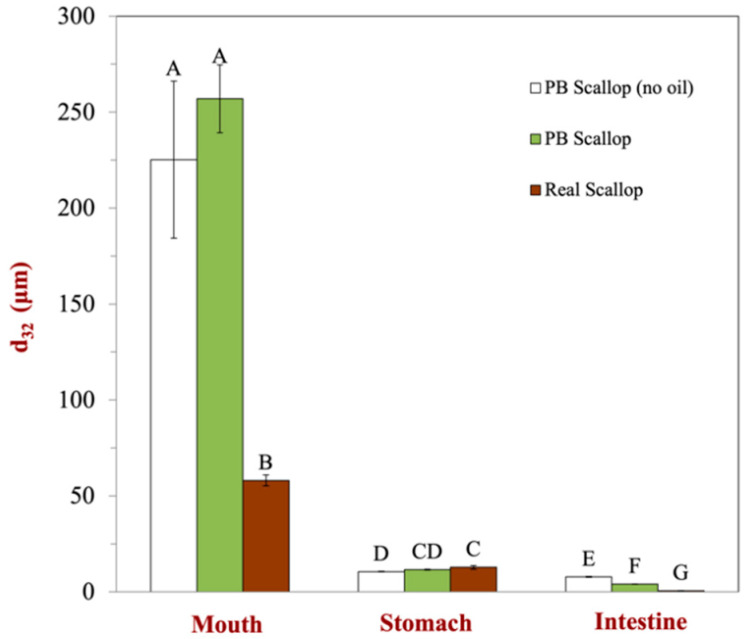
Comparison of impact of gastrointestinal transit on the mean particle diameter (d_32_) of plant-based scallops (with and without flaxseed oil) to that of real scallops. Significant differences (*p* < 0.01) between samples are labeled by various upper-case letters.

**Figure 2:**
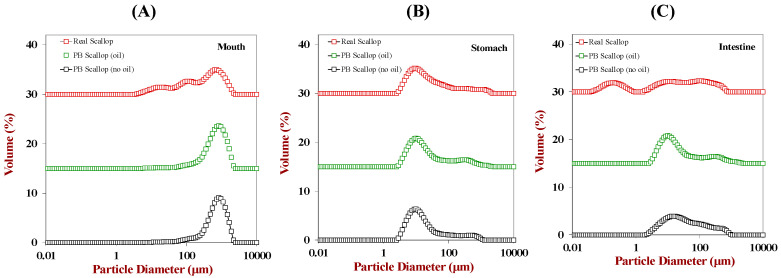
Comparison of the impact of gastrointestinal transit on the particle size distributions of plant-based scallops (with and without flaxseed oil) to real scallops in the oral (**A**), gastric (**B**), and small intestinal (**C**) phases.

As shown in Figure 1 and Figure 2, laser diffraction analysis indicated that the mean particle diameters and particle size distributions of the scallop analogs with and without flaxseed oil in the mouth phase were not significantly different. Thus, the presence of the oil droplets did not have an appreciable impact on the oral behavior of the scallop analogs. The size of the particles in the real scallop was much smaller after exposure to the mouth phase than that in the scallop analogs. The oral fluids and samples were blended for 2 min using a high shear mixer to mimic the disruptive mechanical forces foods experience in the human mouth during mastication. Presumably, the scallop muscle tissue broke down more easily when exposed to simulate oral conditions than the protein–polysaccharide structures in the plant-based scallops, leading to smaller particle sizes.

Following passage through the stomach phase, the particle sizes of both the scallops and scallop analogs exhibited a significant reduction. This size reduction was primarily attributed to the activity of gastric pepsin, which hydrolyzes protein molecules into peptides and amino acids, thereby leading to the breakdown of the overall structure. Notably, there were no major differences in the behavior of the plant-based scallop analogs with and without flaxseed, indicating that the presence of the oil droplets had a minimal impact on the breakdown of the biopolymer matrix under gastric conditions. Interestingly, the particle sizes of the scallops and scallop analogs were quite similar after exposure to the stomach phase, implying that the mechanical, solution, and enzymatic conditions in the gastric fluids facilitated comparable disassembly of their structures.

After the small intestine phase, there was a further reduction in the particle sizes of the scallops and scallop analogs, which can mainly be attributed to protein hydrolysis caused by pancreatic proteases. The scallop analogs containing the oil droplets had a smaller mean particle diameter than the ones without oil droplets. This effect may have been due to the formation of small colloidal particles (mixed micelles and vesicles) during the digestion of the oil droplets, which contributed to the light scattering pattern from which the particle size distribution was estimated. The real scallop had a substantially smaller particle size than the scallop analog after being exposed to small intestinal conditions, which suggests that there may have been a greater amount of protein digestion. Our results are consistent with previous studies, in which the authors also found that the mean particle diameters of plant-based meats are larger than those found in real meats [18], which was attributed to differences in the resistance of the food matrices to disruption and digestion.

### 3.3. Particle Electrical Characteristics

**Figure 3:**
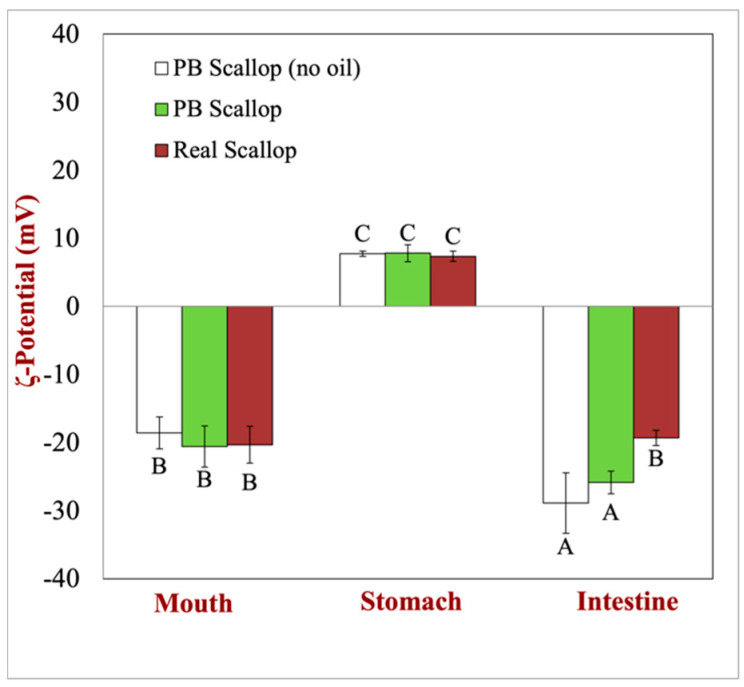
Comparison of gastrointestinal transit on the surface potential (ζ-potential) of plant-based scallops (with and without flaxseed oil) to real scallops. Statistical significance (*p* < 0.01) is represented by the different upper-case letters (A, B, and C) between various samples.

Knowledge of the electrical characteristics of colloidal particles provides insights into their identity and interactions [19]. The electrical characteristics of the colloidal particles in the scallops and scallop analogs were monitored throughout the simulated human gut by measuring changes in their ζ-potential (Figure 3). Within the mouth phase, all samples exhibited a strong negative charge. In the case of real scallops, this can be attributed to their high protein content. Under neutral conditions, the pH surpasses the isoelectric point of the muscle proteins in scallops, resulting in a net negative charge. Additionally, the mucin present in the simulated saliva carries a negative charge under oral conditions, contributing to the electrophoretic signal used for ζ-potential calculations. Similarly, in the case of the scallop analogs, the citrus pectin also possesses a negative charge under neutral oral conditions, further contributing to the overall negative charge.

In the stomach phase, both the scallops and scallop analogs were positively charged. This phenomenon can be primarily attributed to the high protein content present in all the samples and the strongly acidic conditions of the gastric fluids (pH 3), which falls below the isoelectric point of the proteins. Consequently, the proteins exhibited a net positive charge. Interestingly, the plant-based scallop analogs displayed similar ζ-potential values to the real scallops, despite the inclusion of pectin. This could be attributed to two potential reasons. First, the pea protein concentration was much higher than the pectin concentration, and so its electrical characteristics dominated. Second, the carboxyl groups on the pectin molecules would have been partially neutralized under strongly acidic conditions, such that the pK_a_ value of pectin is known to be around 3.5.

In the small intestinal phase, the particles in both the scallops and scallop analogs became strongly negatively charged again. In this region, the negative charge is due to several phenomenon. First, the neutral conditions are above the isoelectric point of the proteins, which leads to a net negative charge on them (and any peptides produced through hydrolysis). Second, there is a range of other negatively charged substances in the small intestine that were either added as part of the gastrointestinal constituents (like bile acids and digestive enzymes) or generated due to lipid or protein digestion (like free fatty acids and peptides). Moreover, the pectin within the scallop analogs is indigestible in the upper GIT. Consequently, it also contributes some negative charge, especially under the neutral conditions in the intestinal fluids, since then the carboxyl groups will be fully deprotonated (-COO^-^). This effect may account for the fact that scallop analogs had higher negative charges than real scallops after small intestine digestion.

### 3.4. Microstructure

**Figure 4:**
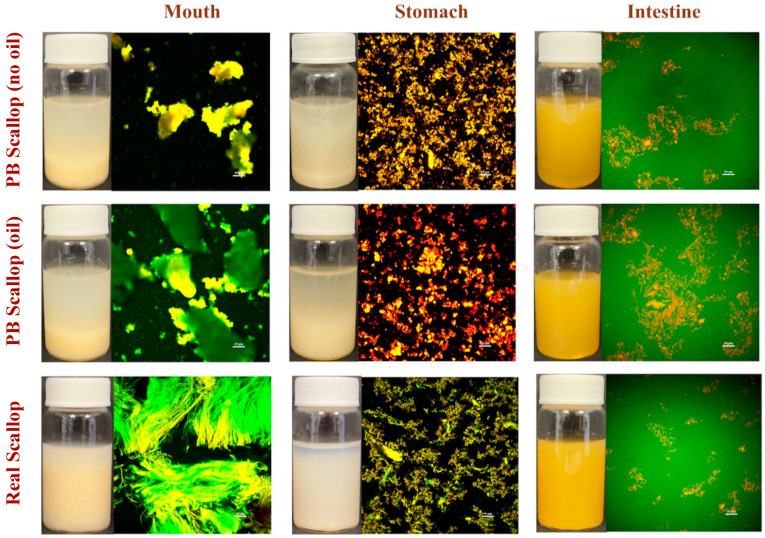
Microstructures of plant-based scallops (with and without flaxseed oil) and real scallops after exposure to mouth, stomach, and small intestine conditions. The proteins were stained green, while the lipids were stained red.

To investigate the spatial distribution of proteins and lipids in the scallops and scallop analogs under simulated gastrointestinal conditions, confocal fluorescence microscopy was employed. Protein staining was achieved using FITC, while lipid staining was achieved using Nile Red (Figure 4).

In the oral phase, large protein-rich chunks were observed in the scallop analogs, while large fibrous structures were observed in the real scallops. These results indicate that there were some similarities and some differences in the structures of the scallops and scallop analogs in the oral phase. The microstructures of the plant-based scallops with and without 0.5% (*w*/*w*) flaxseed oil were fairly similar, which indicates the presence of the oil droplets did not strongly interfere with the breakup of the protein-rich structures during simulated mastication. The differences between the real and plant-based scallops would be expected to cause some differences in the perceived mouthfeel of the two products during mastication. In future studies, it would therefore be useful to compare the sensory attributes of real and plant-based scallops during consumption. If there were appreciable differences, then it would be important to redesign the plant-based scallops to mimic the oral behavior of real scallops more closely.

After entering the stomach phase, the scallops and scallop analogs were broken down into clusters of aggregated particles (Figure 4). As discussed earlier, this is probably due to protein hydrolysis by pepsin, as well as to changes in solution conditions and the application of mechanical forces. There was little difference in the structures of the scallop analogs with and without flaxseed oil, suggesting that the oil droplets did not greatly impact protein digestion and structure breakdown. Fluorescence staining indicated that the oil droplets in the samples containing flaxseed oil appeared to be embedded within the protein matrix. Overall, the structures formed by the real and plant-based scallops after digestion in the stomach were fairly similar.

In the small intestine phase, only small particles were observed in the scallops and scallop analogs, suggesting that most of the large protein-rich structures had been digested. Moreover, the uniform green color in the background suggests that the digested proteins (peptides) were dispersed throughout the aqueous phase. There did appear to be some fibrous materials remaining in the samples after the small intestine phase, especially in the plant-based scallops. These structures may have been due to the presence of undigested pectin, which may have aggregated with other components in the system (such as bile salts and calcium).

Photographs of the different scallop samples showed that their appearance changed throughout the simulated gastrointestinal tract (Figure 4). After the mouth stage, all the samples contained a whitish sediment below a watery serum layer. This sediment most likely contained the protein-rich structures that resulted from the breakdown of the scallops under simulated mastication conditions. After the stomach stage, all the samples still had a whitish sediment below a watery serum layer. Again, this sediment layer is likely to have consisted of partly digested protein-rich fragments that aggregated with each other and moved downwards due to the forces of gravity. After the small intestine phase, all the samples had a uniform yellowish appearance, which is probably because most of the large particles had been broken down and so they were more resistant to gravitational separation. The yellowish color is a result of the bile salts that are added to the simulated small intestinal fluids.

### 3.5. Protein Digestion

**Figure 5:**
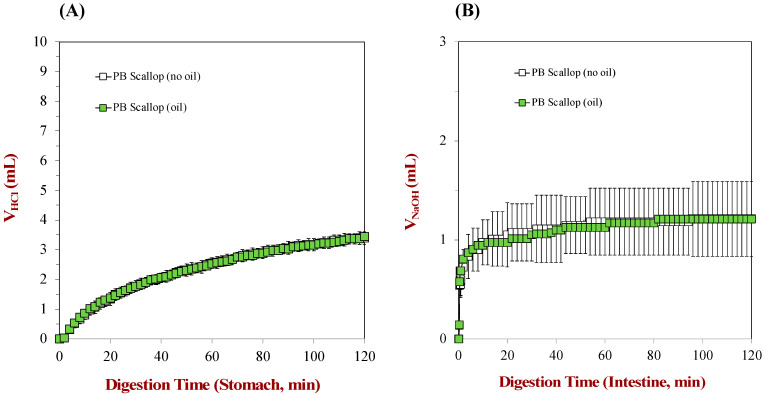
The effect of 0.5 wt% flaxseed oil on the volume of NaOH required to maintain a constant pH during the gastric (**A**) and small intestine (**B**) phases of a simulated gastrointestinal model.

Finally, we measured the digestibility of the proteins in the scallops and scallop analogs using the pH-stat method. Protein digestion mainly occurs within the stomach and small intestine phases due to the activity of gastric and pancreatic proteases. Any lipid digestion would have mainly occurred in the small intestine because we did not include gastric lipase within the simulated gastric fluids. Moreover, considering the relatively low content of fat (<1%) relative to protein (>10%) in both the scallops and scallop analogs, we hypothesized that the lipids would not greatly contribute to the analysis of protein digestion. To verify this hypothesis, the rate and extent of digestion of the scallop analogs were measured in the absence and presence of flaxseed oil droplets using the pH-stat method under stomach and small intestine conditions (Figure 5). The volumes of NaOH solution added to the reaction mixtures to maintain a constant pH were similar for the scallop analogs with and without flaxseed oil. This was true in both the stomach and small intestine phases. These results therefore indicated that the presence of a low concentration of oil droplets in the scallops did not influence protein hydrolysis. Consequently, we ignored the impact of the oil droplets on the calculation of the degree of protein hydrolysis in the subsequent experiments.

**Figure 6:**
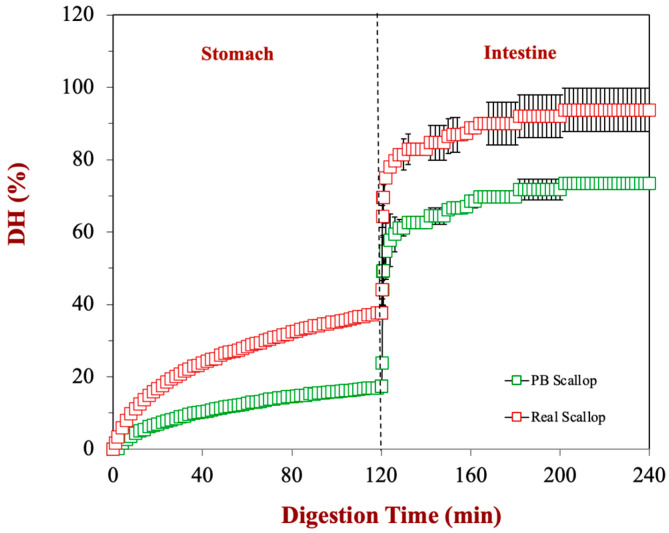
Comparison of protein hydrolysis of plant-based scallops (with 0.5% flaxseed oil) and real scallops during the gastric and small intestinal stages of a simulated GIT model.

Protein digestion in the real and plant-based scallops was then compared under simulated stomach and small intestine conditions (Figure 6). Based on the pH-stat measurements, around 18.8% and 61.4% of protein were digested in the stomach and small intestine phases for the real scallop, respectively, leading to a total protein digestion of around 80.2%. In contrast, around 8.7% and 47.7% of the protein was digested in the plant-based scallop, leading to a total protein digestion of around 56.4%. Thus, the extent of protein hydrolysis in the real scallops was substantially higher than the scallop analogs in both the stomach and intestine phases. It is well known that plant proteins are less digestible than muscle proteins, which has been attributed to a variety of reasons [11,20]. Many plant proteins have a tight globular structure that is held together by multiple disulfide bonds. Moreover, they tend to be less water-soluble than animal proteins. As a result, it is more difficult for the digestive enzymes (proteases) to access the peptide bonds in the polypeptide chains of plant proteins, leading to a slower rate and extent of digestion. Moreover, ingredients derived from plants may contain antinutritional factors, like phytates, tannins, trypsin inhibitors, and lectins, which can retard protein hydrolysis [24,25,26]. Also, the presence of pectin in the scallop analogs can inhibit the digestive processes through several mechanisms, including forming gelled phases that trap macronutrients and make them more difficult for access by digestive enzymes or by binding directly to digestive enzymes and reducing their activity [27,28]. A similar result has been reported in other studies, where it was found that real pork and beef had a higher protein digestibility than plant-based pork and beef analogs [26] and where the proteins in real beef burgers were reported to be more digestible than those in plant-based beef burgers [24]. These results may have important implications for the nutritional quality of plant-based seafood. In the future, it would be useful to perform in vivo feeding studies using animals or humans to ascertain whether protein digestibility was also lower under more realistic gastrointestinal conditions. It should be noted that the digestibility of the real scallop (80.2%) was somewhat less than that reported for other kinds of animal proteins, which is often great than 90% [20,29,30]. This may have occurred for the following several reasons: (i) the mincing process used to simulate mastication did not fully breakdown the scallop tissue; (ii) the scallop tissue had a different structure to that found in meat, egg, or dairy products; (iii) the digestibility of the scallops decreased during storage; (iv) limitations of the simulated gastrointestinal model. Even so, our results still show that the real scallop was digested more than the plant-based version.

## 4. Conclusions and Future Prospects

In this study, we compared the gastrointestinal fate of real scallops with scallop analogs and found that there were some important differences. In particular, the real scallops exhibited a higher protein digestibility than the plant-based versions. This effect was mainly attributed to differences in protein structures, the presence of antinutritional factors, and the impact of pectin on the hydrolysis of the proteins in the plant-based scallop analogs. A lower protein digestibility would mean that people who adopted a diet containing plant-based seafood analogs may obtain less amino acids. Moreover, pea proteins do not contain all the essential amino acids required in the human diet [19]. In particular, they are deficient in the sulfur-containing amino acids methionine and cysteine. In order to avoid the adverse nutritional consequences of switching to a plant-based diet, it would therefore be important to have a higher total protein concentration in plant-based foods than found in the animal-based founds they are designed to replace. Moreover, it may be important to blend plant proteins from different courses, such as those derived from legumes and cereals, so as to obtain the full complement of essential amino acids required. In future studies, it will be important to compare the sensory attributes of real and plant-based scallop analogs, as well as their in vivo gastrointestinal fate, and impact on human health. Careful formulation of plant-based foods would ensure that there are no unforeseen adverse health implications of adopting a more plant-based diet in the future, thereby promoting the transition to a more sustainable food supply.

## Data Availability

Data presented in the study are available upon request to the corresponding author.
